# Reward Devaluation Attenuates Cue-Evoked Sucrose Seeking and Is Associated with the Elimination of Excitability Differences between Ensemble and Non-ensemble Neurons in the Nucleus Accumbens

**DOI:** 10.1523/ENEURO.0338-19.2019

**Published:** 2019-12-10

**Authors:** Meike C. Sieburg, Joseph J. Ziminski, Gabriella Margetts-Smith, Hayley M. Reeve, Leonie S. Brebner, Hans S. Crombag, Eisuke Koya

**Affiliations:** Sussex Neuroscience, School of Psychology, University of Sussex, Falmer BN1 9QG, United Kingdom

**Keywords:** intrinsic excitability, neuronal ensembles, nucleus accumbens, reward devaluation, synaptic physiology

## Abstract

Animals must learn relationships between foods and the environmental cues that predict their availability for survival. Such cue–food associations are encoded in sparse sets of neurons or “neuronal ensembles” in the nucleus accumbens (NAc). For these ensemble-encoded, cue-controlled appetitive responses to remain adaptive, they must allow for their dynamic updating depending on acute changes in internal states such as physiological hunger or the perceived desirability of food. However, how these neuronal ensembles are recruited and physiologically modified following the update of such learned associations is unclear. To investigate this, we examined the effects of devaluation on ensemble plasticity at the levels of recruitment, intrinsic excitability, and synaptic physiology in sucrose-conditioned *Fos-GFP* mice that express green fluorescent protein (GFP) in recently activated neurons. Neuronal ensemble activation patterns and their physiology were examined using immunohistochemistry and slice electrophysiology, respectively. Reward-specific devaluation following 4 d of *ad libitum* sucrose consumption, but not general caloric devaluation, attenuated cue-evoked sucrose seeking. This suggests that changes in the hedonic and/or incentive value of sucrose, and not caloric need, drove this behavior. Moreover, devaluation attenuated the size of the neuronal ensemble recruited by the cue in the NAc shell. Finally, it eliminated the relative enhanced excitability of ensemble (GFP^+^) neurons against non-ensemble (GFP^−^) neurons observed under non-devalued conditions, and did not induce any ensemble-specific changes in excitatory synaptic physiology. Our findings provide new insights into neuronal ensemble mechanisms that underlie the changes in the incentive and/or hedonic impact of cues that support adaptive food seeking.

## Visual Abstract

**Figure:**
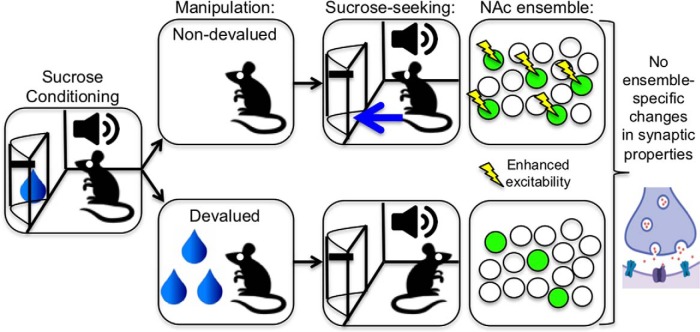

## Significance Statement

Learned associations between food and the cues that predict their availability are encoded in neuronal ensembles in reward-relevant brain areas, such as the nucleus accumbens. Such learning is often accompanied by synaptic and intrinsic plasticity within these ensemble neurons. However, it is unclear how these plasticity changes manifest specifically in cue-activated neurons in response to decreases in reward value [e.g., following reward-specific or general (caloric) devaluation]. We reveal that shifts in excitability, but not excitatory, synaptic physiology between ensemble and non-ensemble neurons in the nucleus accumbens shell coincide with reward-specific devaluation. Our findings provide new insights into how changes in the perceived properties of food reward update cue–food associations by potentially fine-tuning neuronal excitability.

## Introduction

Animals and humans form associations between environmental cues and the foods whose availability they predict (Petrovich, 2013; Jansen et al., 2016). Such cues obtain motivational significance following Pavlovian conditioning and exert powerful control over food seeking (Day and Carelli, 2007; Petrovich, 2013). Critically, organisms have to adapt their appetitive behaviors and related physiological responses not only according to the changing external, but also internal environment. For instance, excessive consumption of a certain type of food can alter its current attractiveness via changes in homeostatic need or its incentive and/or hedonic properties to regulate cue responsivity (Holland and Rescorla, 1975; Goldstone et al., 2009; West and Carelli, 2016). The malfunctioning of such behavioral flexibility may lead to inappropriate responding to food cues and dysregulation of food intake (i.e., overeating) and contribute to excessive weight gain (Boswell and Kober, 2016; Jones et al., 2018; Kosheleff et al., 2018). These are pressing issues in today’s society, in which we are surrounded by cues associated with unhealthy foods (e.g., junk food advertisements). Hence, elucidating the neurobiological processes underlying the updating of cue–food associations is crucial to obtain a better understanding of maladaptive eating behaviors.

It has been shown that associations between cues and rewarding substances such as food and drugs of abuse are dependent on sparsely distributed sets of neurons called neuronal ensembles (Pennartz et al., 1994; Carelli et al., 2000; Koya et al., 2009; Whitaker et al., 2016, 2017; Ziminski et al., 2017, 2018). These neurons can act as memory engrams to encode and store cue reward memory representations (Tonegawa et al., 2015; Whitaker and Hope, 2018). In addition to other mesocorticolimbic structures, these appetitive memory ensembles are found in the nucleus accumbens (NAc), a brain area well established to play a causal role in hedonic processing and incentive learning (Kelley, 2004; Day and Carelli, 2007; Castro et al., 2015; West and Carelli, 2016).

Importantly, intrinsic and synaptic plasticity modulate neuronal network function in the wider mesocorticolimbic network and plays a pivotal role in many forms of associative learning (Stuber et al., 2008; Kourrich et al., 2015; Whitaker et al., 2017). The former primarily involves changes in the electrical or excitability properties of the neuron that influence neuronal firing, while the latter involves changes in neuronal communication at the synapse (Kourrich et al., 2015). For instance, studies using *Fos-GFP* mice that express green fluorescent protein (GFP) in behaviorally activated neurons have shown that intrinsic and synaptic plasticity within NAc ensembles, particularly in the shell region, help to encode cue–reward associations (Barth, 2004; Whitaker et al., 2016; Ziminski et al., 2017). Recently, it was found that changes in appetitive associative strength following extinction learning restricted the ability of food cues to recruit a hyperexcitable neuronal ensemble in the NAc shell subregion (Ziminski et al., 2017). Also, studies have shown that NAc shell neurons activated by specific drug–cue associations exhibit remodeling of excitatory glutamatergic synapses (Koya et al., 2012; Whitaker et al., 2016). Together, physiological modifications in a select group of neurons are likely to establish highly specific appetitive associative memories.

Here, we examined how ensemble-specific changes in intrinsic and synaptic plasticity underlie updating of cue–food associations using a reward-specific devaluation procedure. This approach is widely used to assess behavioral flexibility following changes in the rewarding value of food (West and Carelli, 2016). To this end, we devalued sucrose reward using a reward-specific, sucrose satiation procedure and compared it with a non-reward specific satiation manipulation. Subsequently, we examined plasticity changes in behaviorally activated NAc shell neurons in sucrose-conditioned *Fos-GFP* mice at the levels of ensemble size, excitability, and synaptic physiology following reward-specific devaluation.

## Materials and Methods

### Animals

Male wild-type C57BL/6 mice were purchased from Charles River UK. Male heterozygous *Fos-GFP* mice (https://www.jax.org/strain/014135; RRID:IMSR_JAX:014135) on a C57BL/6 background that originated from the laboratory of Allison Barth (Carnegie Mellon University, Pittsburgh, PA) were obtained from the in-house breeding program at the University of Sussex. All mice were housed two to three per cage and maintained on a 12 h light/dark cycle (lights on at 7:00 A.M.) at a temperature of 21 ± 1°C and 50 ± 5% humidity, and had access to standard chow (BK001 E Rodent Breeder and Grower Diet, SDS) and *ad libitum* water. Unless noted, 1 week before and for the entire duration of the behavioral experiments, mice were food restricted to 90% of their free-feeding body weight (adjusted for age). Mice were 9–10 weeks old at the beginning of behavioral testing. *Fos-GFP* mice were used for experiments examining the effects of devaluation on Pavlovian approach (cue-evoked food seeking), Fos expression, and physiological parameters. These mice condition and exhibit food seeking similarly to wild-type mice (Ziminski et al., 2017*)*. Wild-type mice were used for the experiments examining the effects of caloric satiation on Pavlovian approach. All experiments were conducted during the light phase. All animal procedures were performed in accordance with the regulations of the University of Sussex Animal Welfare and Ethical Review Body (AWERB).

### Behavioral experiments

#### Apparatus

All behavioral procedures were conducted in conditioning chambers (15.9 × 14 × 12.7 cm; Med Associates), each enclosed within a sound-attenuating and light-resistant cubicle. The conditioning chamber was fitted with a recessed magazine situated in the center of one side wall, which dispensed 10% sucrose solution serving as the unconditioned stimulus (US). An infrared beam detected head entries into the magazine. The house light was situated in the side panel and was on for the duration of each training or test session. A mechanical relay served as an auditory (click) conditioned stimulus (CS; Med Associates). Initiation and running of behavioral protocols, including the recording of head entries into the food magazine, was performed using Med-PC IV (Med Associates; RRID:SCR_012156).

#### Behavioral procedures

**Figure 1.**
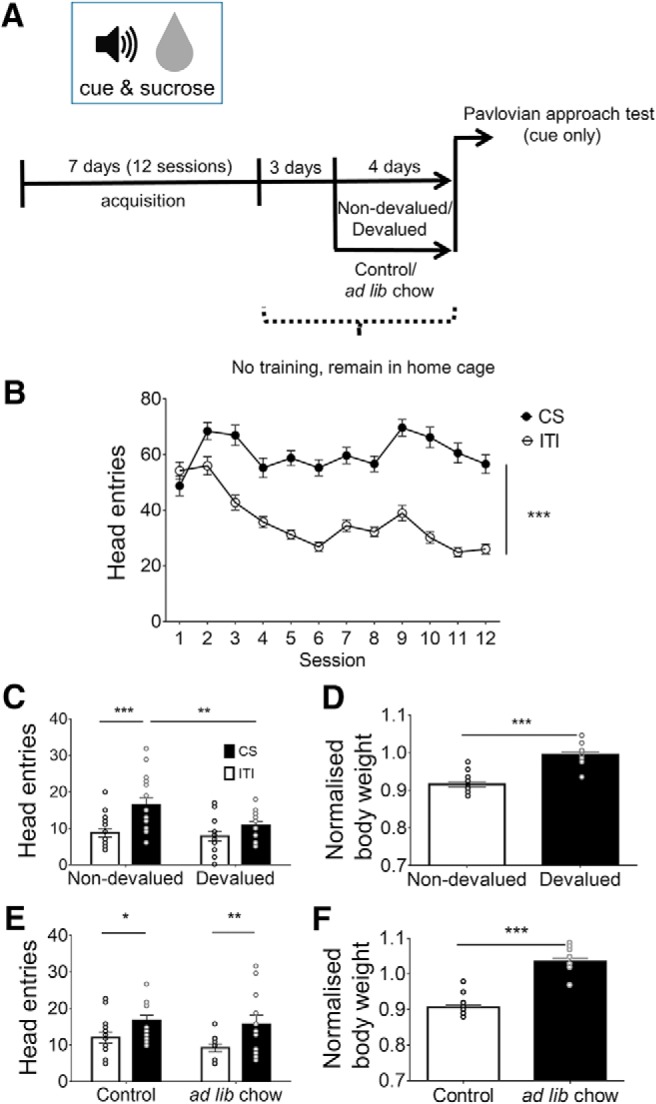
Sucrose reward devaluation, but not caloric satiation, attenuates Pavlovian approach behavior. ***A***, Time line for the Pavlovian approach behavioral paradigm with devaluation and caloric satiation. ***B***, The number of head entries in sucrose delivery magazine during acquisition in response to a sucrose-associated cue (CS) is significantly higher than during ITI; *n* = 32 asterisks indicate the main effect of trial, ***p < 0.001. ***C***, The number of head entries during the Pavlovian approach test in Non-devalued and Devalued mice. Head entries during the cue are significantly higher only in the Non-devalued condition. **p = 0.008, ***p < 0.001. *n* = 14-16/group. ***D***, Body weight normalized to free-feeding body weight in Non-devalued mice is significantly lower than in Devalued mice. ***p < 0.001. *n* = 16 per group. ***E***, No difference in the number of head entries during the Pavlovian approach test during sucrose-associated CS and ITI between *ad libitum* (*ad lib*) chow and Control mice. Head entries during the cue are significantly higher. *p = 0.03, **p = 0.007. *n* = 12–14/group. ***F***, Body weight normalized to free feeding body weight in food-restricted mice is significantly lower than in *ad libitum* chow mice independent of conditioning. ***p < 0.001. *n* = 12–14/group. All values are the mean ± SEM. *Figure Contributions:* M.C.S., J.J.Z., G.M.-S., H.R., and L.S.B. performed experiments; M.C.S. analyzed the data.

Before conditioning, mice underwent a single session of magazine training, which began following the initial head entry into the food magazine. During this session, they received 40 presentations of 10% sucrose solution (∼15 µl) in the food magazine on a random interval (RI) 30 schedule to get accustomed to the sucrose delivery procedure. Starting the next day, mice underwent 11–12 Pavlovian conditioning sessions (on average, 24 min/session; one to two times daily in the morning [8:00 A.M. to 12:00 P.M. (noon)] and/or afternoon [12:00 P.M. (noon) to 5:00 P.M.]) over 7 consecutive days. The illumination of the house light indicated the start of each session, which consisted of six 120 s CS presentations (yoked across conditioning chambers), separated by 120 s RI intertrial interval (ITI) periods. During each 120 s CS period, ∼15 µl of 10% sucrose solution was delivered into the magazine on an RI 30 s schedule. Following conditioning, mice remained in the colony room for 7-9 d until test day. Three days following the final conditioning session (Fig. 1*A*), mice were randomly allocated to one of two groups for the remaining 4–6 d for the following: (1) reward-specific devaluation experiments in which all mice continued to be food restricted, and one group of mice (Devalued group) received *ad libitum* sucrose solution in their home cage, whereas the control (Non-devalued) group received an additional water bottle; and (2) caloric satiation experiments in which one group of mice (*ad libitum* chow group) received *ad libitum* chow in their home cage, whereas the Control group continued to be food restricted until test day. On test day, mice underwent Pavlovian approach testing, to assess cue-evoked sucrose seeking, which consisted of a single session that was similar to the conditioning session, but under extinction conditions (i.e., in the absence of sucrose delivery to avoid the interference of acute sucrose consumption).

### Fos immunohistochemistry

Following testing for Pavlovian approach, mice from the devaluation experiments remained in the conditioning chambers for an additional ∼1 h to allow for optimal Fos expression. Subsequently, they were anesthetized using sodium pentobarbital in saline (1:10; 200 mg/kg, i.p.). Mice were transcardially perfused with ice-cold PBS (concentrations in mm: NaCl 137, KCl 2.7, Na_2_HPO_4_ 10, and KH_2_PO_4_ 1.8, pH 7.4) for 5 min (5 ml/min) and with ice-cold 4% paraformaldehyde (PFA; catalog #158127, Sigma-Aldrich) for 20 min (5 ml/min) using a peristaltic pump (Masterflex L/S, Cole Parmer). Thirty minutes after the end of the perfusion, brains were removed, postfixated in 4% PFA at 4°C for ∼22 h, and then cryoprotected in 30% sucrose solution in PBS for 3–5 d. Brains were frozen on dry ice and stored at −80°C until further use. Brains were sliced into 30 µm coronal sections containing NAc (anteroposterior 1.5 mm from bregma; Paxinos and Franklin, 2012) using a cryostat (Leica CM 1900, Leica Microsystems) and stored in PBS with sodium azide (0.02%) or cryopreservant.

Free-floating slices were washed three times for 10 min in PBS, incubated in 0.3% hydrogen peroxide in PBS for 15–20 min to block endogenous peroxidase activity and subsequently washed three times in PBS. To block nonspecific binding sites and permeabilize cell membranes, slices were incubated in 3% NGST (normal goat serum with Triton X-100; Vector Laboratories) for 1 h. Slices were incubated in primary antibody (1:8000; rabbit anti-c-Fos, sc-52, LOT A2914, Santa Cruz Biotechnology; RRID:AB_2106783) in 3% NGST over night at 4°C. Next, slices were washed three times in PBS and incubated in the secondary antibody (1:600; biotinylated goat anti-rabbit lgG H + L, Vector Laboratories; RRID:AB_2313606) in 1% NGST for 2 h. After three subsequent washes in PBS, slices were incubated in ABC solution (Vector Laboratories; RRID:AB_2336818) for 1 h and then washed twice in PBS. Slices were incubated in 0.04% DAB, 0.05% nickel ammonium sulfate, and 0.04% hydrogen peroxide in PBS for ∼30 min, and washed three times in PBS. Slices were mounted in water onto Fisherbrand Superfrost Slides (Thermo Fisher Scientific) and dried overnight. For dehydration, slides went through the following steps: 2× distilled water on ice for 3 min, 30% ethanol for 2 min, 60% ethanol for 2 min, 90% ethanol for 2 min, 95% ethanol for 2 min, 100% ethanol for 2 min, 100% ethanol for 2 min, and 2× HistoClear (National Diagnostics) for 10 min. Finally, slides were coverslipped using Histomount (National Diagnostics), dried overnight, and stored at room temperature.

Bright-field images of the NAc shell (hereafter, NAc) were taken using a QI click camera (Qimaging) attached to an Olympus BX53 bright-field microscope and iVision-Mac software (version 4.0.15, Biovision Technologies; RRID: SCR_014786). Fos^+^ neurons were counted manually bilaterally in a blind manner at a magnification of 100× using iVision software. Two images were taken per hemisphere (dorsal and ventral), and the numbers of Fos^+^ neurons were added to get one value per hemisphere. Between hemispheres, values were averaged to get one value per animal. Our Fos analysis was restricted to medial portions of the NAc due to low Fos expression in the lateral NAc.

### Electrophysiology

#### *Ex vivo* brain slice preparation

Ninety minutes after the start of Pavlovian approach testing, mice were deeply anaesthetized with ketamine (Anaesktin, Dechra Veterinary Products) and xylazine (Rompun, Bayer Health care) in saline, and then transcardially perfused with ice-cold NMDG solution (in mm): NMDG 93, KCl 2.5, NaH_2_PO_4_ 1.2, NaHCO_3_ 30, HEPES 20, d-glucose 25, C_6_H_7_NaO_6_ 5, SC(NH_2_)_2_ 2, C_3_H_3_NaO_3_ 3, MgSO_4_H_2_0 10, and CaCl_2_.2H_2_0 0.5, with osmolarity of 300–310 mOsm and pH 7.4 (Ting et al., 2018). Following perfusions, the brains were immersed in ice-cold, filtered NMDG solution for 2 min. The cerebellum was removed, and the brain was mounted onto a stage and placed in a slicing chamber filled with ice-cold NMDG solution. Coronal slices 250 µm thick were cut corresponding to ∼1.5 mm anteroposterior from bregma. Slices were stored in NMDG solution for 5 min at 32°C and then transferred to artificial CSF (aCSF) at room temperature until recording. NMDG solution and aCSF (concentrations in mm: NaCl 126, KCl 4.5, MgCl_2_ 1, CaCl_2_ 2.5, NaH_2_PO_4_ 1.2, d-glucose 11, and NaHCO_3_ 26, pH 7.4) were continuously bubbled with a 95% O_2_/5% CO_2_ mixture.

#### Electrophysiological recording

We recorded from NAc shell medium spiny neurons (MSNs), which are the principal neurons of this area using similar criteria as reported in the study by Ziminski et al. (2017). For NAc current-clamp recordings, the slices were hemisectioned and transferred to the recording chamber continuously refilled with aCSF at 32°C (flow rate, ∼2 ml/min). GFP^+^ neurons were identified using a 488 nm laser line from a Revolution XD Spinning Disk Confocal System (Andor) attached to an Olympus BX51W1 microscope (see Fig. 3*B*). Whole-cell patch-clamp recordings were performed using intracellular solution (ICS; concentrations in mm: K-gluconate 125, KCl 10, HEPES 10, MgCl_2_*6H_2_O 2, EGTA 1, CaCl_2_*2H_2_O_2_ 0.1, Mg-ATP 2, and Na-GTP 0.2, at pH 7.25)-filled borosilicate capillary glass pipettes (inner diameter, 0.86 mm; outer diameter, 1.5 mm; resistance 5-7 MΩ; Sutter Instrument) using a P-97 electrode puller (Sutter Instrument). Alexa Fluor 568 dye (100 µm; catalog #A10437, Thermo Fisher Scientific) was added to the ICS to confirm patched neurons by colocalization with GFP. MSNs were identified using morphology, resting membrane potential (RMP), and action potential (AP) waveform, and held at −75 mV for the duration of the recordings. The liquid junction potential was −13.7 mV and was not adjusted for. The current-clamp recording protocol consisted of 800 ms current injections starting at −60 pA and increasing in 4 pA steps.

Data were collected with a Multiclamp 700B amplifier (Molecular Devices), and WinEDR (version 3.7.5) and WinWCP Software (version 5.2.2; courtesy of Dr. John Dempster, University of Strathclyde, Glasgow, UK; http://spider.science.strath.ac.uk/sipbs/software_ses.htm; RRID: SCR_014713). Signals were digitized at 10 kHz and filtered at 5 kHz (PCI 6024E, National Instruments) and low-frequency noise was filtered out using a HumBug (Quest Scientific) module. The input resistance (Ri) was calculated as the slope of the *I–V* curve between −60 and 20 pA injections. Rheobase was calculated manually. Spike kinetics (amplitude and half-width) and afterhyperpolarization (AHP) were calculated using Mini Analysis Software (version 6.0; Synaptosoft; RRID:SCR_002184), and spike counts were calculated using Stimfit 0.14 software (Python 2.7.9; Guzman et al., 2014). The basic membrane properties are summarized in Table 1. The number of GFP^+^ and GFP^−^ neurons recorded per mouse was kept approximately constant at two to four neurons in voltage-clamp recordings and four to six neurons in current-clamp recordings, and the order of recordings was counterbalanced.

**Table 1:** Basic membrane properties from the NAc shell in Non-devalued and Devalued mice

	Non-devalued		Devalued		Interaction	Main effect	
	GFP^−^	GFP^+^	GFP^−^	GFP^+^	GFP × devaluation	GFP	Devaluation
RMP (mV)	−70.8 ± 0.7	−69.4 ± 1.1	−69.1 ± 0.8	−70.3 ± 0.8	*F*_(1,38)_ = 2.28, *p* = 0.14	*F*_(1,38)_ = 0.19, *p* = 0.66	*F*_(1,38)_ = 0.02, *p* = 0.9
Rheobase (pA)	115.0 ± 10.5**	63.2 ± 4.0**	96.7 ± 13.5	91.2 ± 11.9	*F*_(1,37)_ = 4.57, *p* = 0.04	*F*_(1,37)_ = 0.20, *p* = 0.66	*F*_(1,37)_ = 7.02, *p* = 0.01
Input resistance (MΩ)	151.2 ± 10.7***	246.5 ± 27.6***	160.2 ± 12.0	200.6 ± 17.2	*F*_(1,37)_ = 1.28, *p* = 0.26	*F*_(1,37)_ = 13.04, *p* < 0.01	*F*_(1,37)_ = 0.34, *p* = 0.57
AHP (mV)	−8.9 ± 0.5	−9.8 ± 0.8***	−7.7 ± 0.8	−7.3 ± 0.8***	*F*_(1,38)_ = 0.78, *p* = 0.38	*F*_(1,38)_ = 0.07, *p* = 0.79	*F*_(1,38)_ = 6.07, *p* = 0.02
AP half- width (ms)	1.4 ± 0.1**	1.8 ± 0.15**,***	1.4 ± 0.03	1.4 ± 0.04***	*F*_(1,37)_ = 2.9, *p* = 0.1	*F*_(1,37)_ = 6.0, *p* = 0.02	*F*_(1,37)_ = 4.31, *p* = 0.04
AP amplitude (mV)	67.4 ± 1.9	58.7 ± 4.2	65.9 ± 3.3	63.5 ± 3.8	*F*_(1,37)_ = 0.82, *p* = 0.37	*F*_(1,37)_ = 2.53, *p* = 0.12	*F*_(1,37)_ = 0.22, *p* = 0.64

Data in first four columns are expressed as the mean ± SEM.

*p < 0.05, **p < 0.01, *post hoc* comparison GFP^+^ vs GFP^−^; ***p < 0.05, *post hoc* comparison Non-devalued vs Devalued.

Voltage-clamp recordings were conducted in the presence of the GABA_A_ receptor channel blocker picrotoxin (100 μm; Sigma-Aldrich) using the following ICS (in mm): spermine 0.1, CsCH_3_SO3 120, NaCl 5, TEA-Cl 10, HEPES 10, EGTA 1.1, MgATP 4, Na-GTP 0.3, and QX314 4.6 (Lidocaine, Sigma-Aldrich). Spontaneous EPSCs (sEPSCs) were analyzed over a 30 s period. Responses were evoked through bipolar stimulating electrodes (CBASD75, FHC), within 400 μm of the neuron with 0.1 ms pulses at 0.033 Hz. Series resistance was monitored using −10 mV voltage steps (100 ms), and only neurons maintaining stable access (<15% change) were included in the analyses. Paired-pulse ratios (PPRs) were calculated by stimulating twice in succession and dividing second peak by the first peak (average of triplicate) across ITIs of 20, 40, 60, 80, 100, 150, and 200 ms. AMPA receptor/NMDA receptor (AMPAR/NMDAR) current ratios were calculated from the averages of 10–20 evoked EPSCs at +40 mV with and without d-APV (NMDA receptor antagonist, 50 μm; Hello Bio). For each neuron, the AMPAR current (with d-APV) was subtracted from the combined current (without d-APV) to yield the NMDAR current (Koya et al., 2012). The AMPAR current peak was divided by the NMDAR current peak to yield AMPAR/NMDAR current ratios. AMPAR rectification curves were produced by averaging triplicate stimulations at −80, −60, −40, −20, 0, 20, and 40 mV in the presence of d-APV. The AMPAR rectification index was calculated by dividing the EPSC peak amplitude at −80 mV by the peak amplitude at +40 mV. The ratio of the chord conductance (G = *I–V*) was calculated by dividing the chord conductance at +40 mV by the chord conductance at −80 mV (G_+40 mV_/G_−80 mV_). Traces in figures have stimulus artifacts removed.

### Experimental design and statistical analysis

Data were analyzed and visualized using GraphPad Prism 6 (GraphPad software, RRID:SCR_002798), SPSS (IBM SPSS statistics; RRID:SCR_002865), and Excel (Microsoft). Spontaneous EPSCs were analyzed using Mini Analysis Software (version 6.0; Synaptosoft; RRID:SCR_002184), whereas evoked EPSCs (e.g., PPRs) were analyzed using WinWCP Software. Statistical analyses are summarized in Table 2. All data are presented as the mean ± SEM. Data points exceeding ±2 SDs or greater from the mean were excluded from the analyses. Group data are presented as the mean ± SEM. ANOVAs were followed up by Fisher’s least significant difference test.

**Table 2:** Summary of statistical analyses

	Data structure	Type of test	95% Confidence interval																																								
Check for pre-existing differences in acquisition of Pavlovian conditioning between (future) groups: Devalued vs Non-devalued and *ad libitum* chow vs control	Quantification of head entries during acquisition of Pavlovian conditioning during CS and ITI, displayed as difference score	Two-way mixed ANOVAs	Session		1		2			3		4				5			6							7			8						9		10		11			12	
			Non-devalued		0.14–0.02		0.0069–0.16			0.18–0.30		0.15–0.33				0.24–0.40			0.28–0.40							0.19–0.35			0.21–0.35						0.22–0.38		0.29–0.41		0.34–0.44			0.30–0.46	
			Devalued		-0.25–0.055		0.014–0.29			0.13–0.27		0.033–0.25				0.25–0.39			0.29–0.45							0.18–0.32			0.17–0.35						0.21–0.39		0.31–0.49		0.41–0.51			0.25–0.41	
			Control		-0.28–0.059		-0.0060–0.21			0.057–0.26		0.0019–0.22				0.074–0.25			0.053–0.27							0.14–0.38			0.18–0.40						0.24–0.32		0.13–0.25		0.21–0.35			0.23–0.37	
			*Ad libitum* chow		-0.066–0.088		0.030–0.19			0.22–0.34		0.044–0.28				0.11–0.25			0.20–0.34							0.16–0.34			0.18–0.34						0.19–0.33		0.17–0.35		0.23–0.35			0.11–0.35	
Acquisition of Pavlovian conditioning (Fig. 1*B*)	Quantification of head entries during acquisition of Pavlovian conditioning during CS and ITI	Two-way repeated measures ANOVA	Session		1		2			3	4				5		6		7							8						9					10		11			12	
			CS		41.34–56.04		62.12–74.76			58.39–72.25	48.13–62.31				53.28–64.16		49.29–61.15		55.64–66.48							50.85–62.33						65.36–76.70					59.76–71.30		53.28–67.84			49.93–59.59	
			ITI		46.08–56.86		50.13–61.81			37.22–48.16	31.68–39.76				27.73–34.59		23.32–30.24		29.49–37.29							28.78–35.60						33.18–44.58					25.32–32.56		20.90 -27.16			22.41–29.53	
Pavlovian approach test (Fig. 1*C,E*)	Quantification of head entries during Pavlovian approach test during CS and ITI in Devalued vs Non-devalued and *ad libitum* chow vs control groups	Two-way mixed ANOVAs											CS																		ITI												
			Non-devalued										12.28–20.60																		6.35–11.27												
			Devalued										8.61–13.11																		5.20–8.80												
			Control										13.68–19.74																		8.73–15.27												
			*Ad libitum* chow										9.90–21.26																		6.84–11.50												
Body weights (Fig. 1*D,F*)	Body weights normalized to free feeding body weight in Devalued vs Non-devalued and *ad libitum* chow vs control groups	Unpaired two-tailed t-tests	Non-devalued																		0.91–0.93																						
			Devalued																		0.99–1.01																						
			Control																		0.89–0.91																						
			*Ad libitum* chow																		1.01–1.05																						
Fos quantification (Fig. 2*B*)	Quantification of Fos^+^ cells in NAc shell in Devalued and Non-devalued groups; two images were taken per hemisphere (dorsal and ventral) and numbers of Fos^+^ neurons were added to get one value per hemisphere, between hemispheres values were averaged to get one value per mouse	Two-tailed *t* test	Non-devalued																		58.01–98.83																						
			Devalued																		39.83–64.97																						
Excitability data (Fig. 3*C–F*)	Quantification of action potentials after injection of increasing current steps (20–116 pA) in GFP^+^ and GFP^−^ neurons in Devalued and Non-devalued groups	Three-way mixed ANOVAs, two-way mixed ANOVAs						Current step (pA)					20			32		44					56				68			80						92		104			116		
			Non-devalued					GFP^−^					0.0–0.0			0.0–0.0		0.0–0.0					0.0–0.0				0.0–0.0			0.0–0.0						-0.41–4.41		-0.20–7.80			1.53–11.27		
								GFP^+^					0.0–0.0			0.0–0.0		0.0–0.0					-0.21–0.87				2.35–8.01			5.25–13.65						8.42–18.12		10.48–21.52			11.95–24.77		
			Devalued					GFP^−^					0.0–0.0			0.0–0.0		0.0–0.0					-0.15–0.37				-0.71–3.51			1.07–8.93						2.32–12.22		2.92–14.36			4.16–15.48		
								GFP^+^					0.0–0.0			0.0–0.0		0.0–0.0					-0.38–3.78				-0.59–8.19			-0.66–11.46						1.32–15.28		3.62–18.78			6.38–22.02		
*I–V* curves (inlays Fig. 3*C–F*)	Voltage displacement (mV) to subthreshold current injections in the range of (−60 to −20 pA) in GFP^+^ and GFP^−^ neurons in Devalued and Non-devalued groups	Three-way mixed ANOVAs, Two-way mixed ANOVAs				−60		−56		−52	−48			−44		−40		−36		−32			−28		−24		−20	−16		−12			−8	−4		0	4	8	12	16		20	
			Non-devalued	GFP^−^		−8.59 to −4.92		−8.39 to −6.02		−7.82 to 5.62	−7.52to −5.35			−7.01 to −5.04		−6.40 to −4.50&		−6.03 to −4.32		−5.21 to −3.61			−4.89 to −3.34		−4.36 to −3.03		−3.84 to −2.48	−3.84 to −2.48		−2.30 to −1.50			−1.49 to −0.99	−0.83 to −0.49		−0.049 to 0.61	1.08–1.70	1.58–2.71	2.21–3.28	2.90–4.39		3.80–5.37	
				GFP^+^		−**13.6 to** −**9.3**		−**13.6 to** −**8.8**		−**12.4 to** −**8.1**	−**11.7 to** −**7.6**			−**11.0 to** −**7.1**		−**9.9 to** −**6.4**		−**9.60 to** −**6.14**		−**8.47 to** −**5.32**			−**7.47 to** −**4.80**		−**6.95 to** −**4.17**		−**6.13 to** −**3.47**	−**5.18 to** −**2.95**		−**4.11 to** −**2.37**			−**2.73 to** −**1.49**	−**1.80 to** −**0.40**		−**0.30 to** −**1.18**	**1.11–2.13**	**1.89–4.02**	**3.004–4.56**	**3.61**− **6.26**		**4.95–10.081**	
			Devalued	GFP^−^		−**8.45 to** −**6.88**		−**7.90 to** −**6.31**		−**7.48 to** −**5.97**	−**7.78 to** −**5.85**			−**7.31 to** −**5.37**		−**6.46 to** −**4.73**		−**5.70 to** −**4.54**		−**5.69 to** −**3.96**			−**4.60 to** −**3.54**		−**4.10 to** −**3.19**		−**3.56 to** −**2.85**	−**2.73 to** −**2.08**		−**2.81 to** −**1.82**			−**1.42 to** −**0.99**	−**1.18 to** −**0.22**		−**0.32 to 0.70**	**0.88–1.92**	**1.96–2.53**	**2.46**–**4.09**	**2.66–4.09**		**3.66–5.12**	
				GFP^+^		−**11.510 to** −**8.13**		−**11.75 to** −**7.70**		−**11.056 to** −**7.33**	−**1.005 to** −**7.058**			−**9.102 to** −**6.49**		−**8.09 to** −**5.73**		−**8.28 to** −**5.59**		−**7.088 to** −**4.76**			−**6.53 to** −**4.28**		−**5.29 to** −**3.53**		−**4.52 to** −**3.18**	−**4.25 to** −**2.67**		−**3.18 to** −**2.12**			−**1.72 to** −**1.21**	−**1.056 to 0.46**		−**0.16 to 1.10**	**1.07–1.75**	**1.87–3.52**	**2.71 – 4.50**	**3.79 – 5.54**		**4.90 – 7.73**	
Membrane and AP parameters (Fig. 4*A–F*, Table 1)	RMP, input resistance, AHP, AP amplitude and half-width, Rheobase in GFP^+^ and GFP^−^ neurons in Devalued and Non-devalued groups	Two-way ANOVAs	RMP						Non-devalued						GFP^−^									−72.30 to −69.30																			
															GFP^+^									−71.82 to −66.90																			
									Devalued						GFP^−^									−70.88 to −67.30																			
															GFP^+^									−72.15 to −68.45																		
			Input resistance						Non-devalued						GFP^−^									127.02–175.28																			
															GFP^+^									185.04–308.02																			
									Devalued						GFP^−^									131.78–170.12																			
															GFP^+^									161.81–239.41																		
			AHP						Non-devalued						GFP^−^									−10.02 to −7.82																			
															GFP^+^									−11.49 to −8.09																			
									Devalued						GFP^−^									−9.60 to −5.88																			
															GFP^+^									−9.18 to −5.38																		
			AP amplitude						Non-devalued						GFP^−^									67.83–72.03																			
															GFP^+^									49.43–67.99																			
									Devalued						GFP^−^									58.67–73.17																			
															GFP^+^									54.98–72.08																		
			AP half-width						Non-devalued						GFP^−^									1.17–1.61																			
															GFP^+^									1.42–1.94																			
									Devalued						GFP^−^									1.29–1.43																			
															GFP^+^									1.35–1.51																		
			Rheobase						Non-devalued						GFP^−^									91.26–138.74																			
															GFP^+^									54.17–72.23																			
									Devalued						GFP^−^									61.02–120.98																			
															GFP^+^									64.20–118.20																		
AMPAR/NMDAR current ratio (Fig. 5*A*)	Ratios of AMPAR to NMDAR currents (recorded at +40 mV) in GFP^+^ and GFP^−^ neurons in Devalued and Non-devalued groups	Two-way ANOVAs	Non-devalued																			GFP^−^													0.88–2.24								
																						GFP^+^													0.93–1.37								
			Devalued																			GFP^−^													0.95–1.59								
																						GFP^+^													0.95–1.23								
AMPAR rectification index (Fig. 5*B*)	Absolute ratios of AMAR EPSC recorded at −80 mV to the EPSC recorded at +40 mV in GFP^+^ and GFP^−^ neurons in Devalued and Non-devalued groups	Two-way ANOVA	Non-devalued																			GFP^−^													2.91–4.43								
																						GFP^+^													3.11–3.97								
			Devalued																			GFP^−^													1.88–5.98								
																						GFP^+^													1.65–4.73								
Chord conductance ratios	Chord conductance (G = *I–V*) at +40 mV was divided by the chord conductance at −80 mV in GFP^+^ and GFP^−^ neurons in Devalued and Non-devalued groups		Non-devalued																			GFP^−^													0.44–0.68								
																						GFP^+^													0.50–0.64								
			Devalued																			GFP^−^													0.32–0.84								
																						GFP^+^													0.39–1.01								
sEPSC frequency (Fig. 5*C*)	Number of sEPSCs over a 30 s period expressed in Hz in GFP^+^ and GFP^−^ neurons in Devalued and Non-devalued groups	Two-way ANOVA	Non-devalued																			GFP^−^													2.37–6.59								
																						GFP^+^													2.63–5.83								
			Devalued																			GFP^−^													1.66–3.42								
																						GFP^+^													1.60–3.48								
sEPSC amplitude (Fig. 5*C*)	Mean amplitude of sEPSCs over a 30 s period expressed in Hz in GFP^+^ and GFP^−^ neurons in Devalued and Non-devalued groups	Two-way ANOVA	Non-devalued																			GFP^−^													14.05–18.59								
																						GFP^+^													14.77–19.91								
			Devalued																			GFP^−^													16.73–19.31								
																						GFP^+^													15.07–19.57								
Paired-pulse ratios (Fig. 5*D*)	Ratio of second to first evoked EPSC over interstimulus intervals of 20, 40, 60, 80, 100, 150, and 200 ms in GFP^+^ and GFP^−^ neurons in Devalued and Non-devalued groups	Three-way mixed ANOVA										ISI					20					40				60				80					100			150				200	
			Non-devalued									GFP^−^					1.01–1.29					1.11–1.51				0.96–1.28				0.86–1.34					0.83–1.41			0.86–1.20				0.65–1.51	
												GFP^+^					0.92–1.34					0.89–1.37				0.94–1.42				0.90–1.42					0.93–1.19			0.89–1.11				0.90–1.10	
			Devalued									GFP^−^					0.96–1.56					0.92–1.36				0.93–1.35				0.93–1.29					0.84 -1.22			0.88–1.06				0.84–1.06	
												GFP^+^					0.90–1.40					0.73–1.81				0.88–1.52				0.70–1.70					0.93–1.19			0.56–1.34				0.25–1.27	

#### Behavioral data

The total number of head entries into the sucrose delivery magazine during acquisition were analyzed using a two-way repeated-measures ANOVA including cue presentation (ITI, CS) and session (1–12) as within-subjects factors. Two-way mixed ANOVAs were used to test for pre-existing differences in a Pavlovian approach, using session (1–12) as within-subjects factor and caloric satiation (control, *ad libitum* chow) or devaluation (Non-devalued, Devalued) as between-subjects factor. The test data were analyzed using two-way mixed ANOVAs using cue presentation (ITI, CS) as a within-subjects factor and devaluation (Non-devalued, Devalued) or caloric satiation (Control, *ad libitum* chow) as a between-subjects factor. Body weights were analyzed using unpaired two-tailed *t* tests. A total of four mice from the *ad libitum* chow and Devalued groups were excluded from the test analyses due to equipment malfunction.

#### Fos expression

Fos quantification data were analyzed using a two-tailed *t* test comparing the number of Fos^+^ neurons per square millimeter between Non-devalued and Devalued conditions. Brain sections from two mice were damaged and could not be used for cell quantification.

#### Electrophysiology

Spike counts and *I–V* curves were first analyzed using a three-way mixed ANOVA with devaluation (Non-devalued, Devalued) and GFP (+/*–*) as between-subjects factors, and current step as the within-subjects factor. This was followed up by two-way mixed ANOVAs using current step as a within-subjects factor and GFP (+/*–*) or devaluation (Non-devalued, Devalued) as a between-subjects factor.

RMP, rheobase, Ri, AHP, spike amplitude, and half-width were analyzed using two-way ANOVAs with devaluation (Non-devalued, Devalued) and GFP (+/*–*) as between-subject factors. sEPSC frequency and amplitude, and AMPAR rectification index were analyzed using two-way ANOVAs with devaluation (Non-devalued, Devalued) and GFP (+/–) as between-subjects factors. The ratio of G = *I–V* at +40 mV over −80 mV (G_+40 mV_/G_−80 mV_) was analyzed using a one-sample *t* test against the population mean of 1, which indicates a lack of rectification (Bonferroni corrections were used to control for multiple comparisons). PPRs were analyzed using a three-way mixed ANOVA with devaluation (Non-devalued, Devalued) and GFP (+/–) as between-subjects factors and interstimulus interval as a within-subjects factor. AMPAR/NMDAR current ratios and sEPSC parameters were analyzed using a two-way ANOVA with devaluation (Non-devalued, Devalued) and GFP (+/–) as between-subjects factors.

## Results

### Acquisition of Pavlovian conditioning

We assessed the establishment of a cue–sucrose association following 12 sessions of Pavlovian conditioning, during which an auditory cue (clicker) was repeatedly paired with 10% sucrose solution delivery (Fig. 1*A*). With conditioning, mice made a significantly greater number of head entries into the sucrose delivery magazine during the CS period (cue and sucrose presentation) versus the non-CS/ITI period; this difference was mainly due to a progressive decrease in responding during the ITI as conditioning progressed (Fig. 1*B*). A two-way repeated-measures ANOVA revealed a significant interaction of cue presentation (CS, ITI) and session (*F*_(11,341)_ = 18.12, *p* < 0.0001), and significant main effects of cue presentation (*F*_(1,31)_ = 321, *p* < 0.0001) and session (*F*_(11,341)_ = 9.957, *p* < 0.0001). This finding indicates that mice learned the association between the cue and sucrose delivery.

### Reward-specific devaluation attenuates Pavlovian approach

Seven days after the last acquisition session and after 4–6 d of either *ad libitum* chow or sucrose solution in the home cage, mice underwent Pavlovian approach testing under extinction conditions (Fig. 1*A*).

We first assessed the effect of sucrose devaluation on Pavlovian approach. A two-way mixed ANOVA showed a significant interaction of cue presentation × devaluation (*F*_(1,28)_ = 5.275, *p* = 0.0293) and a significant effect of cue presentation (*F*_(1,28)_ = 27.84, *p* < 0.0001). *Post hoc* group differences, indicating a reduction of cue-evoked sucrose seeking in Devalued mice, are presented in Figure 1*C*. Importantly, no pre-existing differences between groups were detected during acquisition (interaction of devaluation × session: *F*_(11,330)_ = 0.6798, *p* = 0.7577; session: *F*_(11,330)_ = 26.67, *p* < 0.0001; devaluation: *F*_(1,30)_ = 0.016, *p* = 0.9002).

Frequent sucrose consumption results in weight gain (Te Morenga et al., 2012). Thus, as a measure for sucrose consumption, we measured the body weights of Devalued mice following *ad libitum* sucrose consumption and compared them with those of Non-devalued mice. A *t* test (*t*_(30)_ = 8.629, *p* < 0.0001) revealed that mice in the Devalued group exhibited significantly higher body weights than their Non-devalued counterparts (Fig. 1*D*), indicating that mice in the Devalued group consumed a significant amount of sucrose.

### Caloric satiation does not modulate Pavlovian approach

Next, we assessed whether increased caloric consumption alone would result in reduced cue reactivity. To this end, we trained an additional group of mice using the same behavioral procedure as above, but instead of sucrose we provided them with *ad libitum* access to chow in their home cage. Caloric satiation did not modulate cue-evoked sucrose seeking (Fig. 1*E*), but cue presentations increased the number of head entries during the CS, as shown by a two-way ANOVA (interaction cue presentation × caloric satiation: *F*_(1,24)_ = 0.3335, *p* = 0.569; cue presentation: *F*_(1,24)_ = 14.26, *p* = 0.0009; caloric satiation: *F*_(1,24)_ = 1.081, *p* = 0.3089). *Post hoc* comparisons are shown in Figure 1*E*. Again, no pre-existing differences between groups were detected during acquisition (interaction caloric satiation × session: *F*_(11,308)_ = 0.8548, *p* = 0.5853; session: *F*_(11,308)_ = 10.54, *p* < 0.0001; caloric satiation: *F*_(1,28)_ = 0.907, *p* = 0.3491). Also, similar to *ad libitum* sucrose consumption, *ad libitum* chow consumption also increased body weight (*t*_(26)_ = 10.62, *p* < 0.001; Fig. 1*F*). This suggests that cue-evoked sucrose seeking was not attenuated by caloric need alone.

### Devaluation attenuates NAc Fos expression

**Figure 2.**
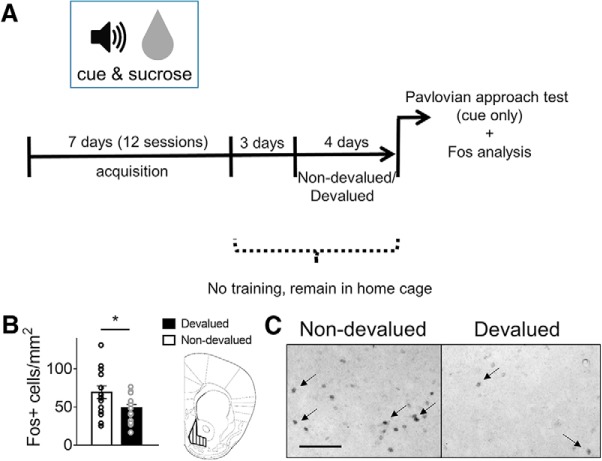
Fos expression in the NAc shell. ***A***, Time line for Pavlovian approach behavioral paradigm with devaluation and subsequent Fos analysis. ***B***, Reward-specific devaluation decreased the Fos expression. *N* = 14/group, *p < 0.05. ***C***, Representative images of Fos staining in NAc shell in the Non-devalued and Devalued groups. All values are the mean ± SEM. Arrows indicate exemplary Fos^+^ cells. Scale bar, 100 µm. Schematic overview was modified after Paxinos and Franklin (2012). *Figure Contributions:* M.C.S. performed experiments and analyzed the data.

Next, we assessed the effects of reward-specific devaluation on neuronal ensemble activity in the NAc by examining the number of Fos-expressing neurons (Fig. 2*A*). A *t* test revealed a significant reduction in Fos^+^ neurons in NAc (*t*_(27)_ = 2.376, *p* = 0.0249) in the Devalued group compared with the Non-devalued group, indicating that a smaller ensemble was recruited in the NAc following reward-specific devaluation (Fig. 2*B*,*C*).

### Devaluation is associated with lack of excitability differences between ensemble and non-ensemble neurons

**Figure 3.**
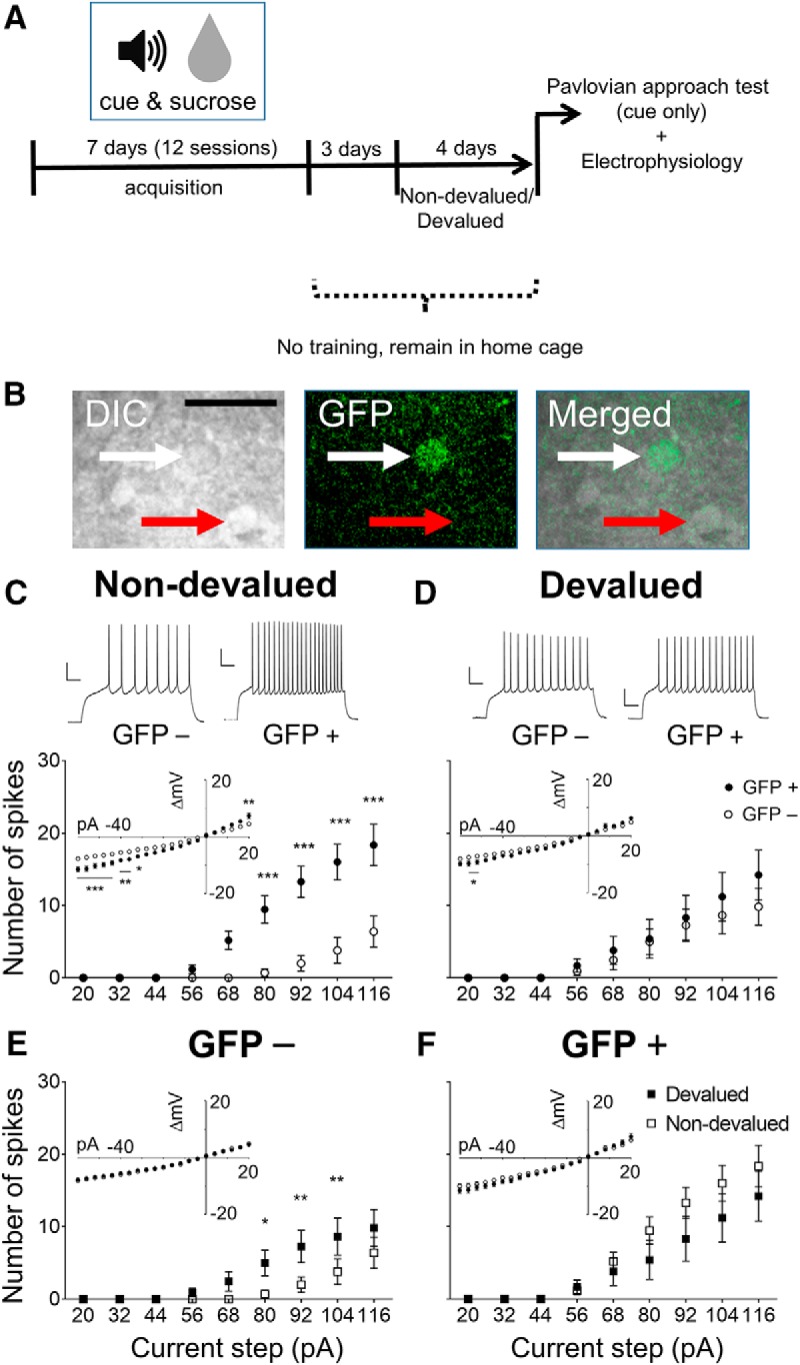
The increased excitability of GFP^+^ neurons compared with surrounding GFP^−^ neurons in NAc shell is attenuated by reward devaluation. ***A***, Time line for the Pavlovian approach behavioral paradigm with devaluation. ***B***, Differential interference contrast (DIC) optics and confocal microscopy (GFP) were used to identify GFP^+^ (white arrow) and GFP^−^ (red arrow) neurons. Scale bar, 20 µm. ***C***, In the Non-devalued group, GFP^+^ cells exhibit increased spiking in response to increasing current injections compared with surrounding GFP^−^ cells. The *I–V* curve (inlay) for GFP^+^ cells are shifted in positive and negative current steps, but not in the intermediate range (GFP^−^, *n* = 10/6; GFP^+^, *n* = 11/6). Representative traces from injections at 116 pA (top). ***D***, After sucrose devaluation, there is no difference in firing capacity between GFP^+^ and GFP^−^ cells. Only a mild downward shift is observed for the *I–V* curves (inlay) from GFP^+^ and GFP^−^ cells (GFP^−^, *n* = 11/9; GFP^+^, *n* = 11/8). Representative traces from injections at 116 pA (top). ***E***, GFP^−^ cells exhibit an increased number of spikes after sucrose devaluation. ***F***, There is no difference in firing capacity or *I–V* curves (inlay) in GFP^+^ cells between the Devalued and Non-devalued groups. *p < 0.05, **p < 0.01, ***p < 0.001. All values are the mean ± SEM, and values to the right of GFP^−^ and GFP^+^ denote the number of cells recorded/number of mice used. Calibration: 20 mV, 100 ms. *Figure Contributions:* M.C.S. performed experiments; M.C.S. and J.J.Z. analyzed the data.

In a separate cohort of mice, we assessed the excitability of cue-responsive, GFP^+^ ensemble and surrounding GFP^−^ non-ensemble MSNs 90 min following the initiation of Pavlovian approach testing (Fig. 3*A*). We injected increasing amounts of current into the neurons and quantified the number of action potentials fired in response to assess the firing capacity of these neurons (Fig. 3). A three-way mixed ANOVA showed an interaction of current step × devaluation × GFP (*F*_(8,304)_ = 3.115, *p* = 0.002), an interaction of current step × GFP (*F*_(8,304)_ = 6.784, *p* < 0.0001), as well as a significant main effect of current step (*F*_(8,304)_ = 53.88, *p* < 0.0001) and GFP (*F*_(1,38)_ = 8.364, *p* = 0.006), but not devaluation (*F*_(1,38)_ = 0.012, *p* = 0.912). To determine what is driving this three-way interaction, we further conducted a two-way ANOVA comparing the firing rates (spike counts) of GFP^+^ and GFP^−^ neurons within Non-devalued mice separately. This revealed an interaction of current step × GFP (*F*_(8,152)_ = 11.84, *p* < 0.0001), as well main effects of current step (*F*_(8,152)_ = 35.64, *p* < 0.0001) and GFP (*F*_(1,19)_ = 18.57, *p* = 0.0004; Fig. 3*C*). This indicates that in Non-devalued mice, GFP^+^ and GFP^−^ neurons differed significantly in firing capacity. A similar ANOVA comparing GFP^+^ and GFP^−^ neurons within the Devalued group yielded a main effect of current step (*F*_(8,152)_ = 21.43, *p* < 0.0001), but no effect of GFP (*F*_(1,19)_ = 0.3584, *p* = 0.5565) or interaction (*F*_(8,152)_ = 0.5413, *p* = 0.8239; Fig. 3*D*). Hence, in the Devalued group, GFP^+^ and GFP^−^ neurons did not differ in firing capacity. *Post hoc* tests are indicated in Figure 3, *C* and *D*. Together, these results indicate that differences in excitability between GFP^+^ and GFP^−^ neurons are eliminated following reward-specific devaluation.

**Figure 4.**
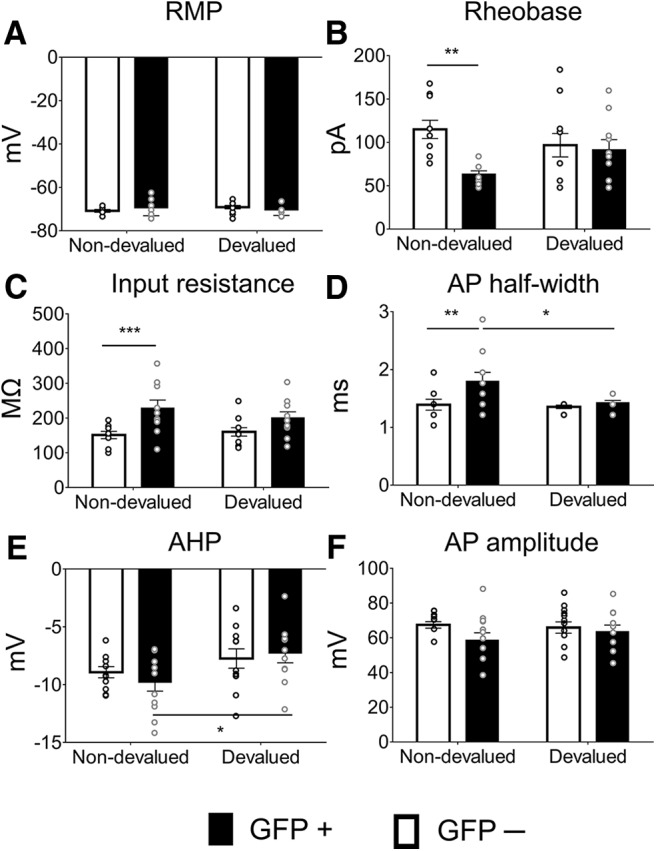
Basic passive membrane and action potential parameters in GFP^+^ and GFP^−^ cells with and without devaluation. ***A***, RMP was unchanged by devaluation or ensemble identity. ***B***, Rheobase was lower in GFP^+^ compared with GFP^−^ cells without devaluation; **p = 0.0047 (Non-devalued: GFP^−^, *n* = 9/5; GFP^+^, *n* = 10/6; Devalued: GFP^−^, *n* = 11/9; GFP^+^, *n* = 10/8). ***C***, Input resistance was specifically increased in GFP^+^ cells without devaluation; **p = 0.0021 (Non-devalued: GFP^−^, *n* = 10/6; GFP^+^, *n* = 10/6; Devalued: GFP^−^, *n* = 11/9; GFP^+^, *n* = 10/8). ***D***, AP half-width was specifically increased in Non-devalued GFP^+^ neurons; *p = 0.0103, **p = 0.0052 (Non-devalued: GFP^−^, *n* = 10/6; GFP^+^, *n* = 11/6; Devalued: GFP^−^, *n* = 10/9; GFP^+^, *n* = 10/8). ***E***, AHP was unchanged by devaluation or ensemble identity. ***F***, AP amplitude was unchanged by devaluation or ensemble identity. All values are the mean ± SEM, values to the right of GFP^−^ and GFP^+^ denote the number of cells recorded/number of mice used, asterisks indicate *post hoc* comparisons after two-way ANOVAs. *Figure Contributions:* M.C.S. performed experiments; M.C.S. analyzed the data.

Excitability changes in both ensemble and non-ensemble neurons underlie alterations in appetitive learning (Whitaker et al., 2017; Ziminski et al., 2017, 2018). Therefore, we compared the spike counts of GFP^+^ and GFP^−^ neurons separately across conditions. For the GFP^−^ non-ensemble neurons (Fig. 3*E*), we discovered an interaction of current step × devaluation (*F*_(8,152)_ = 2.048, *p* = 0.0444) and a main effect of current step (*F*_(8,152)_ = 15.91, *p* < 0.0001), but no main effect of devaluation (*F*_(1,19)_ = 3.271, *p* = 0.0864). *Post hoc* analysis revealed a slight, but significant, increase in spike number in GFP^−^ neurons from the Devalued group, which was not accompanied by any changes in the *I–V* curves or any of the active and passive membrane properties (Figs. 3, 4). For the GFP^+^ ensemble (Fig. 3*F*), two-way mixed ANOVAs revealed no significant interaction of current step × devaluation (*F*_(8,152)_ = 1.33, *p* = 0.2324) or main effect of devaluation (*F*_(1,19)_ = 1.152, *p* = 0.2966), but did reveal a significant main effect of current step (*F*_(8,152)_ = 38.45, *p* < 0.0001). These findings indicate that a slight increase in excitability in GFP^−^ non-ensemble neurons contributed to the lack of excitability differences between the GFP^+^ and GFP^−^ neurons as a function of reward-specific devaluation.

Analysis of *I–V* curves with a three-way mixed ANOVA did not reveal an interaction of current step × GFP × devaluation (*F*_(20,780)_ = 1.212, *p* = 0.236), but did reveal a significant interaction of current step × GFP (*F*_(20,780)_ = 11.031, *p* < 0.0001), as well as a significant effect of current step (*F*_(20,780)_ = 430.768, *p* < 0.0001) and GFP (*F*_(1,39)_ = 16.829, *p* < 0.0001), but not of devaluation (*F*_(1,39)_ = 0.789, *p* = 0.38). To determine what is driving these effects, further analysis using a two-way ANOVA comparing GFP^+^ and GFP^−^ neurons separately within Non-devalued and Devalued groups was conducted. It revealed a significant interaction of current step × GFP (*F*_(20,360)_ = 7.951, *p* < 0.0001) as well as main effects of each factor (current step: *F*_(20,360)_ = 185.5, *p* < 0.0001; GFP: *F*_(1,18)_ = 11.5, *p* = 0.0033) in the Non-devalued group (Fig. 3*C*, inlay), which was similar to the effect observed in the number of spikes. *Post hoc* comparisons between GFP^+^ and GFP^−^ neurons in negative and positive potential are indicated in Figure 3*C* (inlay). In the Devalued group, a two-way ANOVA comparing GFP^+^ and GFP^−^ neurons yielded an interaction of current step × GFP (*F*_(20,380)_ = 2.931, *p* < 0.0001) as well as a main effect of both factors (current step: *F*_(20,380)_ = 217.6, *p* < 0.0001; GFP: *F*_(1,19)_ = 4.504, *p* = 0.0472; Fig. 3*D*, inlay). *Post hoc* tests are indicated in the Figure 3*D* inlay. Similar to our previous analysis of excitability, we next conducted additional two-way ANOVAs in GFP^+^ or GFP^−^ neurons between the Devalued and Non-devalued groups. For both GFP^+^ and GFP^−^ neurons, no significant interaction or effect of devaluation but an effect of current step (GFP^+^: *F*_(20,360)_ = 177.5, *p* < 0.0001, GFP^−^: *F*_(20,380)_ = 267.7, *p* < 0.0001) were revealed (Fig. 3*E*,*F*, inlays). In summary, the differences in the *I–V* curves of GFP^+^ and GFP^−^ neurons seen before devaluation were still present afterward, but were less pronounced and restricted to negative potentials.

To investigate the source of the differences in firing capacity, we examined the RMP, rheobase, Ri, AHP, and AP half-width and amplitude of GFP^+^ and GFP^−^ neurons from the Non-devalued and Devalued groups using two-way ANOVAs (Fig. 4, Table 1). For rheobase (*F*_(1,37)_ = 4.57, *p* = 0.0392; Fig. 4*B*), but for none of the remaining parameters, we found a significant interaction of devaluation × GFP. *Post hoc* testing revealed decreased rheobase in GFP^+^ neurons compared with GFP^−^ neurons in the Non-devalued, but not in the Devalued group. This suggests that devaluation eliminated the differences in the minimum amount of current needed for spiking between ensemble and non-ensemble neurons (Table 1). We only found a main effect of GFP for Ri (*F*_(1,38)_ = 13.47, *p* = 0.0007; Fig. 4*C*) and AP half-width (*F*_(1,37)_ = 6.004, *p* = 0.012; Fig. 4*D*). There was a main effect for devaluation for AHP (*F*_(1,38)_ = 6.07, *p* = 0.02; Fig. 4*E*), AP half-width (*F*_(1,37)_ = 4.31, *p* = 0.04; Fig. 4*D*), and rheobase (*F*_(1,37)_ = 7.02, *p* = 0.01; Fig. 4*B*). *Post hoc* tests are indicated in Figure 4 and Table 1. We did not reveal any effects on RMP and AP amplitude (Fig. 4*A*,*F*). Hence, devaluation did not modulate these properties in an ensemble-specific manner.

### Devaluation does not modulate synaptic properties in an ensemble-specific manner

**Figure 5.**
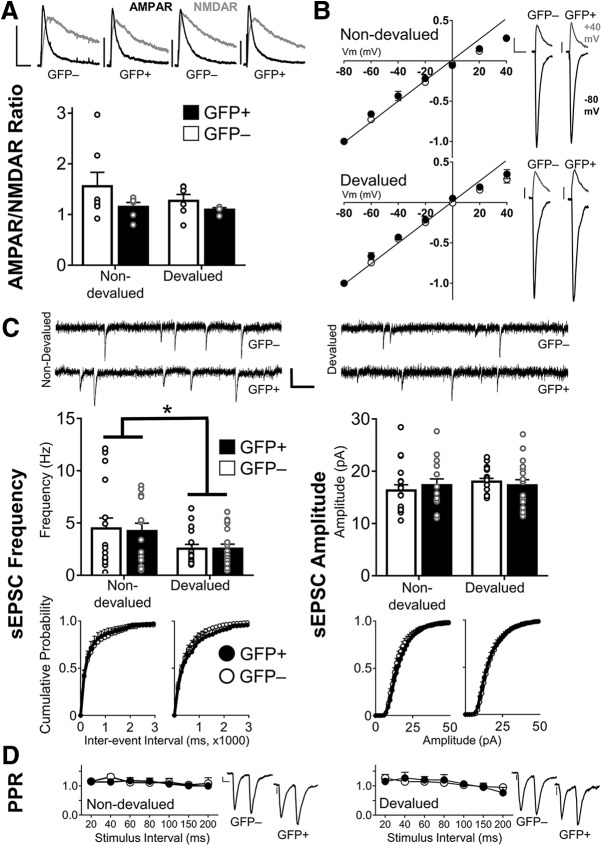
Devaluation did not modulate the synaptic strength of GFP^+^ neurons. ***A***, AMPAR/NMDAR ratios between GFP^+^ and GFP^−^ neurons were similar in both the Non-devalued and Devalued groups (Non-devalued: GFP^−^, 7/7; GFP^+^, 6/5; Devalued: GFP^−^, 6/6; GFP^+^, 4/3). Top, Representative AMPAR/NMDAR traces from GFP^+^ and GFP^−^ neurons. Calibration: 50 pA, 50 ms. ***B***, AMPAR rectification was similar in activated ensembles following non-devaluation and devaluation (Non-devalued group: GFP^−^ =, 5/4; GFP^+^, 4/4; Devalued group: GFP^−^, 5/4; GFP^+^, 5/3). The data shown are normalized to the current peak at −80 mV. Right, Representative images of Non-devalued and Devalued rectification curves in GFP^+^ and GFP^−^ neurons at +40 mV (gray) and −80 mV (black). Calibration: 50 pA, 10 ms. ***C***, Representative sEPSC traces from Non-devalued and Devalued mice. Calibration: 20 pA, 100 ms. sEPSC frequency (left) and amplitude (right) were not selectively modulated in GFP^+^ and GFP^−^ neurons (Non-devalued: GFP^−^, 19/8; GFP^+^, 15/8; Devalued: GFP^−^, 17/6; GFP^+^, 18/6). However, reward devaluation reduced sEPSC frequency nonselectively across both neuron types (*p < 0.05). ***D***, Paired-pulse ratios were similar in GFP^+^ and GFP^−^ neurons from Non-devalued and Devalued mice (Non-devalued: GFP^−^, 13/10; GFP^+^, 8/8; Devalued: GFP^−^, 8/7; GFP^+^, 5/4). Calibration: 100 pA, 10 ms. Data are expressed as the mean ± SEM; values to the right of GFP^−^ and GFP^+^ denote the number of cells recorded/number of mice used. *Figure Contributions:* M.C.S. and J.J.Z. performed experiments; J.J.Z. analyzed the data.

We next investigated the synaptic properties of GFP^+^ and GFP^−^ neurons in Non-devalued and Devalued groups. We first measured the synaptic strength in these neurons by assessing the AMPAR/NMDAR ratios. A two-way ANOVA did not reveal a significant interaction of devaluation × GFP (*F*_(1,19)_ = 0.35, *p* = 0.56; Fig. 5*A*), indicating a lack of differences in synaptic strength across ensembles and conditions. The insertion of GluA2-lacking AMPARs enhances excitatory transmission, and neurons expressing these receptors display inward rectification (Cull-Candy et al., 2006). Therefore, we measured rectification of AMPAR EPSC by dividing the EPSC amplitude at −80 mV by the amplitude at +40 mV in the presence of the NMDA antagonist APV. We observed no significant interaction of GFP × devaluation (*F*_(1,15)_ = 0.37, *p* = 0.55; Fig. 5*B*), indicating no differences in the expression of GluA2-lacking AMPARs across ensembles and conditions.

Previous studies have shown that food restriction and palatable food consumption increase the expression of GluA2-lacking AMPARs in the nucleus accumbens (Oginsky et al., 2016; Ouyang et al., 2017). As such, we examined whether inward rectification was generally present in Devalued and Non-devalued mice that underwent both food restriction and repeated sucrose consumption during training. We calculated the ratio of G at +40 over −80 mV (G_+40 mV_/G_−80 mV_). If rectification is present, then this value is <1. A one-sample *t* test against a population of mean of 1 revealed that in the Devalued group, GFP^+^ neurons did not display rectification (0.70 ± 0.11; *t*_(4)_ = 2.67, *p* = 0.0559), but was observed in GFP^−^ neurons (0.58 ± 0.09; *t*_(4)_ = 4.48, *p* = 0.0110). Also, rectification was observed in GFP^+^ and GFP^−^ neurons in the Non-devalued group (GFP^+^: 0.57 ± 0.02, *t*_(3)_ = 20.16, *p* = 0.0003; GFP^−^: 0.56 ± 0.04, *t*_(4)_ = 10.32, *p* = 0.0005). Collectively, these data suggest that devaluation did not modulate synaptic strength and AMPA receptor function on NAc ensembles. However, these data suggest that we observed widespread expression of GluA2-lacking AMPARs, as indicated by rectification in GFP^−^ non-ensemble neurons regardless of devaluation.

Next, we examined the sEPSC frequency and amplitude. We observed no significant interaction of GFP × devaluation in sEPSC frequency (*F*_(1,65)_ = 0.03, *p* = 0.85; Fig. 5*C*) or amplitude (*F*_(1,65)_ = 0.71, *p* = 0.40; Fig. 5*C*). There was a main effect of devaluation for sEPSC frequency (*F*_(1,65)_ = 6.46, *p* < 0.05), suggesting a generalized decrease in sEPSC frequency in Devalued mice (Fig. 5*C*). Finally, we observed no interaction or main effects in presynaptic release probability as measured using the PPR (GFP × devaluation × interstimulus interval: *F*_(6,180)_ = 0.53, *p* = 0.78), suggesting that the group differences in sEPSC frequency were not driven by presynaptic adaptations (Fig. 5*D*).

## Discussion

Here we examined the effects of devaluation on ensemble plasticity at the levels of recruitment, excitability, and synaptic physiology in sucrose-conditioned *Fos-GFP* mice. After conditioning, we provided mice with 4 d of *ad libitum* access to sucrose or standard chow. Sucrose access, but not caloric satiation alone, attenuated cue-evoked sucrose seeking and hence led to devaluation. This reward-specific devaluation (1) reduced the size of the behaviorally activated NAc shell neuronal ensemble and (2) eliminated differences in excitability between ensemble and non-ensemble neurons that were observed under Non-devalued conditions. Interestingly, devaluation did not alter any ensemble-specific synaptic alterations. Our findings provide new insights into how changes in the rewarding properties of food modulate cue-evoked sucrose seeking by potentially modifying the background excitability of NAc non-ensemble neurons.

### Implications and mechanisms of reduced cue-evoked sucrose seeking and ensemble size following devaluation

Reward-specific devaluation, but not general caloric satiation alone, decreased cue-evoked sucrose seeking. Hence, the incentive and/or hedonic properties of sucrose, but not homeostatic need, may control this behavioral change. The incentive properties relate to the inclination to seek food, whereas the hedonic properties relate to the pleasurable properties associated with food consumption (Castro et al., 2015). One possibility then is that *ad libitum* access to sucrose decreased the incentive properties of the sucrose-associated cue. In support, selective satiation reduces breakpoints on a progressive ratio appetitive task (Baxter et al., 2000). Alternatively, mice in our study may have updated the reward representation according to the new and less attractive value and adapted their food seeking because sucrose overconsumption led to decreases in palatability or hedonic properties (Thompson et al., 1976; Strickland et al., 2018). To directly determine the factors that decreased sucrose seeking, a future study incorporating sucrose consumption and orofacial reactivity during a sucrose consumption test would be needed (Berridge et al., 1981; Johnson et al., 2009; Castro et al., 2015).

Devaluation decreased NAc Fos expression consistent with the role of NAc in mediating the hedonic and incentive properties of sucrose and associated cues (Kelley et al., 1996; Taha, 2005; Cacciapaglia et al., 2012). At the circuit level, neuronal activation after devaluation may be reduced via inhibition from local interneurons that control ensemble size. Additionally, decreased excitatory drive from cortical afferents mediating goal-directed behaviors from areas such as the basolateral amygdala and ventral hippocampus may contribute (Taverna et al., 2005; Wilson, 2007; Shiflett and Balleine, 2010; Stefanelli et al., 2016; LeGates et al., 2018). The result is reduced output into areas such as the lateral hypothalamus and ventral tegmental area, and thus attenuation of cue-evoked sucrose seeking (Kelley et al., 2005; Castro et al., 2015; Yang et al., 2018).

NAc neurons expressing either the dopamine 1 receptor (D_1_R) or dopamine 2 receptor (D_2_R) project to different mesocorticolimbic structures and play distinct roles in reward-related behaviors (Smith et al., 2013). Here, we did not distinguish neurons based on their D_1_R/D_2_R expression. It has recently been observed that conditioning and extinction learning do not modulate the proportion of D_1_R- and D_2_R-expressing ensembles following cue exposure (Ziminski et al., 2017). Also, there are no clear differences in goal-directed behavior on optogenetic stimulation of either subpopulation (Natsubori et al., 2017). Hence, it is likely that devaluation recruits an ensemble with similar levels of D_1_R- and D_2_R-expressing neurons. However, additional investigations are necessary to confirm this.

### Implications for lack of ensemble excitability differences following devaluation

Following reward-specific devaluation, the previous excitability differences observed between ensemble and non-ensemble neurons were eliminated. *In vivo*, such shifts in excitability may modulate neuronal firing in response to cue presentations. In support, devaluation reduces the number of phasically firing NAc neurons in response to sucrose cues (West and Carelli, 2016). But what is the identity of this ensemble activated following devaluation that does not differ in excitability from non-ensemble neurons? After devaluation, we may have recorded from a smaller subset of the same ensemble that was activated under Non-devalued conditions during sucrose seeking, which may have updated the cue–reward association. Alternatively, others have reported that ensembles that promote and inhibit food seeking coexist in the same brain area (Suto et al., 2016; Warren et al., 2016). Therefore, after devaluation we may have recorded from a different and incidentally smaller ensemble, which represented the changed reward value. While distinguishing these two possibilities is challenging, future studies may longitudinally monitor cue-activated NAc neurons with and without devaluation and functionally interrogate them using optogenetics/chemogenetics to determine which of the above possibilities are relevant.

The elimination of excitability differences between ensemble and non-ensemble neurons following devaluation arose from a slight enhancement of excitability only in non-ensemble neurons. These excitability differences are thought to boost the signal-to-noise ratio of information processing of ensemble neurons (Nicola et al., 2000; Ziminski et al., 2018), and its elimination may thus attenuate the responsivity to food-associated cues following devaluation. The cause for this increased background excitability is unclear, but we note that sucrose consumption increases NAc shell dopamine transmission (Roitman et al., 2008). This dopamine release resulting from daily sucrose consumption may enhance MSN excitability through D_1_R activation (Hernández-López et al., 1997). Here, we did not observe any associated changes in active and passive membrane properties in these non-ensemble neurons. This observed lack of change may have resulted from not distinguishing our NAc MSNs based on dopamine receptor expression, which may have masked any subtle cell-type specific changes. Finally, enhancements in firing capacity have been observed following D_1_R activation without any changes in Ri, spike threshold, and duration (Tseng and O’Donnell, 2004), despite the known role of D_1_R activation enhancing L-type Ca^+2^ currents that regulate repetitive firing (Hernández-López et al., 1997). This indicates that subtle changes in passive and active membrane properties may not always be detected despite alterations in firing capacity. Further studies are required to parse out the cellular and intrinsic factors that resulted in this minor, but widespread enhancement in neuronal firing following devaluation.

### Potential reasons for lack of learning- or devaluation-induced ensemble-specific differences in synaptic physiology

Surprisingly, despite the role of glutamate synapse alterations in appetitive learning, we found no alterations in sEPSC frequency and amplitude, AMPAR/NMDAR current ratio, AMPA rectification index, and PPR. We, however, observed a generalized reduction in sEPSC frequency, indicating synaptic alterations induced by *ad libitum* sucrose consumption. This contrasts with studies using drug rewards demonstrating increased spine dynamics in NAc ensembles selectively activated in response to drug-associated cues (Singer et al., 2016; Whitaker et al., 2016). This difference between natural and drug rewards in their ability to generate synaptic alterations in NAc may be due to natural rewards being less potent at eliciting behavioral and neurophysiological changes (Grimm et al., 2003; Chen et al., 2008; Gipson et al., 2013). Additionally, for associative learning paradigms using natural reinforcers, an extended time frame or paradigms with more CS–US pairings may be needed to induce synaptic alterations (Cifani et al., 2012; Guegan et al., 2013a,b; Counotte et al., 2014). Together, the lack of indices of plasticity at glutamatergic synapses demonstrate neuronal ensembles in NAc that may reflect inherent differences of natural and drug rewards and the way their behavioral outcomes are manifested.

### The role of ensemble changes in intrinsic excitability, but not synaptic physiology

Few studies to date have examined the role of both intrinsic and synaptic plasticity in appetitive associative learning. So far, fear conditioning studies have demonstrated the concomitant alterations of intrinsic excitability and synaptic physiology following associative learning (Rosenkranz and Grace, 2002). In contrast, we found neuronal excitability, but not excitatory synaptic physiology, to be altered by devaluation. In line with our findings, previous studies have reported excitability changes independently of synaptic plasticity (Egorov et al., 2002; Labno et al., 2014). It is proposed that alterations in excitability may serve as a transient priming mechanism for initial associative memory formation before synaptic changes take place (Moyer et al., 1996; Janowitz and Van Rossum, 2006; Mozzachiodi and Byrne, 2010). Further research is needed to determine whether our observed excitability changes constitute a transient priming mechanism active during rule learning of the updated reward value and whether synaptic alterations consolidating this updated value might be detectable later on.

### Limitations and conclusion

Reward-specific devaluation, but not caloric satiation, attenuated cue-evoked sucrose seeking. Thus, it is conceivable that the associated effects on Fos expression and ensemble excitability are due to a decreased value of sucrose reward. However, the present study cannot rule out the possibility that our observed Fos and excitability alterations were modulated by caloric satiety provided during sucrose devaluation. Therefore, although caloric satiation alone did not attenuate sucrose seeking, it would be critical in future studies to determine whether caloric satiation attenuates Fos expression and eliminates excitability differences between ensemble and non-ensemble neurons in the absence of CS exposure.

Fos expression requires sustained neuronal activity and therefore only labels strongly activated neurons, which play a role in cue-evoked behaviors (Koya et al., 2009; Cruz et al., 2013; Warren et al., 2016; Whitaker et al., 2016). In *Fos-GFP* rats and mice, GFP is coexpressed with Fos and peaks 2 h after induction and is back to baseline by 24 h (Barth, 2004; Cifani et al., 2012; Koya et al., 2012). Hence, it is unlikely that many of the GFP^+^ neurons in the current study were activated long before the Pavlovian approach test, although GFP^+^ neurons might have been activated by other events close in time. Thus, in our Devalued group, recent sucrose consumption may have induced Fos (Sheng and Greenberg, 1990; Cruz et al., 2015). However, Fos induction in the striatum habituates rapidly, and the consumption of a sweet solution has been shown to not alter Fos expression in NAc (Duncan et al., 1996; Struthers et al., 2005). Hence, our GFP^+^ neurons likely represent neurons activated during Pavlovian approach testing rather than recent sucrose consumption. However, to establish this possibility we would need to use strategies that would label neurons activated by both recent sucrose consumption and CS exposure. Activity-sensitive immediate early genes *homer1a* and *arc* may be useful for such studies as they are used to label neurons activated by distinct stimuli presented at two different time points (Grosso et al., 2015).

Differences in Fos induction based on satiety state have been observed previously*. Ad libitum* chow-maintained rats exhibited no change in NAc Fos protein or mRNA on consumption of a sweet solution or pellets (Duncan et al., 1996; Gao et al., 2017). However, when mice are food restricted, palatable food consumption has been shown to increase Fos expression in NAc (Latagliata et al., 2018). In the current study, we did not see this satiety-based increase in Fos, as after 4 d of sucrose consumption the effects of reward devaluation on Fos expression may outweigh the satiety effects of sucrose consumption, resulting in the observed decrease in Fos levels. To shed light on this, future studies could investigate Fos levels after shorter periods of sucrose consumption.

In this study, all of our mice were trained under “Paired” conditions in which CS and US presentations occurred in temporal proximity. We did not use an “Unpaired” control group that receives CS and US presentations at disparate times (e.g., CS in the conditioning chamber, US in the home cage) to prevent their association. This control group is used to parse out neuronal activation and excitability patterns that are induced by general stimuli that are not explicitly paired with the US. We observed enhanced excitability in CS-activated neurons in our Non-devalued control group. Ziminski et al. (2017) demonstrated in *Fos-GFP* mice that sucrose-associated CSs increased GFP expression by 1.4-fold and recruited a hyperexcitable GFP^+^ ensemble in the Paired group compared with the Unpaired group. These additional GFP^+^ neurons likely represent those that are recruited by sucrose cue exposure. Thus, the ensemble hyperexcitability in the Non-devalued control group occurred as a result of the CS being paired with sucrose and is not a general property of activated neurons. Interestingly, Fos expression decreased by 1.4-fold following devaluation (Fig. 2*B*), which suggests that devaluation reduced Fos expression related to sucrose cue exposure. However, it remains to be determined whether *ad libitum* sucrose consumption alone is capable of attenuating Fos expression in Unpaired mice.

As Devalued mice made fewer head entries during the CS, they may have experienced a reduced amount of extinction learning compared with Non-devalued mice. These differences in extinction learning may have elicited devaluation-independent consequences on NAc activation patterns and hence decreased Fos expression. However, Ziminski et al. (2017) demonstrated that extinction learning decreased NAc *Fos* expression. As Non-devalued mice with more opportunity for extinction learning expressed more Fos than Devalued mice, this reduction is unlikely due to the reduced opportunity to engage in extinction learning in Devalued mice.

Here we revealed that devaluation was associated with altered ensemble size and intrinsic excitability, but not synaptic plasticity in behaviorally activated neuronal ensembles in the NAc shell. Our findings reveal novel mechanisms underlying cognitive and behavioral flexibility. However, future studies are required to elucidate the functional role of devaluation-activated neuronal ensembles. For instance, chemogenetic or optogenetic approaches using *Fos-tTA* mice that allow tagging and stimulation of Fos-expressing neurons will allow us to reveal whether activation of Fos-expressing neurons following devaluation is sufficient to reduce cue-evoked sucrose seeking (Cruz et al., 2013). Additionally, we need to identify the afferent brain areas that regulate these forms of ensemble plasticity and the downstream areas that are modulated as a result to further elucidate mechanisms that suppress food seeking. Such processes are important to understand why certain individuals are hypersensitive to food cues and resistant to internal signals that help limit food intake. 

## Synthesis

Reviewing Editor: Yavin Shaham, NIDA-IRP/NIH Decisions are customarily a result of the Reviewing Editor and the peer reviewers coming together and discussing their recommendations until a consensus is reached. When revisions are invited, a fact-based synthesis statement explaining their decision and outlining what is needed to prepare a revision will be listed below. The following reviewer(s) agreed to reveal their identity: Yan Dong, Yonatan Kupchik.

The reviewers were very positive about the study and only raised several minor comments that can be seen in their review below. We do not think additional experiments are necessary and all is required is to address the reviewers' comments with text changes.

Note: there is no need to change the reporting of the statistical results in the revision,

REVIEWER 1

This is a very interesting study demonstrating that Fos-positive neurons in the nucleus accumbens exhibited increased membrane excitability after sucrose seeking training compared to Fos-negative neurons, while this differential effect was abolished in animals with a potential devaluation procedure. The concept in this manuscript is novel and exciting. The data appear to be solid, and conclusions are reasonable. The quality and innovation of this study are consistent with the scope of eNeuro.

Below are a few minor points for the authors to consider:

1) The devaluation procedure is straightforward and effective. It is also overly simplified in terms of the devaluation procedure as well as the behavioral verification of this procedure. One of the potential caveats is that it is not clear whether the procedure hits the rewarding vs. motivational aspects of sucrose seeking. While additional experiments are not requested, some touch-ups in the overall wording should be considered.

2) Fig. 2: Some additional results or explanations will help the reader to understand whether the GFP-positive neurons in devaled mice are sucrose-sensitive neurons or just randomly labeled neurons through basal activities.

3) Fig. 3: data support the argument that the differences between GFP+ and GFP- neurons are eliminated after devaluation. However, the data also show a relatively higher excitability in GFP- neurons after devaluation (E). This result may lead to alternative interpretations, and should at least be discussed.

4) p57: it may read better if the sentence just finishes by “unhealthy food”. It is not always true the unhealthy food is low in nutrition (in some cases can be extremely high).

REVIEWER 2

The manuscript examines whether seeking for sucrose after a cue is driven by the rewarding value of sucrose or by satiety signals and tries to detect the physiological changes occurring in neuronal ensembles in the NAc that may explain the motivation to consume sucrose after an associated cue is presented. The authors show very nicely that sucrose seeking is driven by the rewarding value of sucrose and not satiety signals and that devaluation of sucrose reward is accompanied by changes in the excitability of ensemble neurons and non-ensemble neurons in the NAc.

The manuscript is very well written and presents new and important data that would contribute to the understanding of the mechanisms driving mice (and probably people...) to consume palatable and rewarding foods after being exposed to a cue. I only have some minor comments/suggestions -

1) The use of spermine in the intracellular solution - the authors indicate that spermine was present in all voltage clamp experiments. This makes sense for measuring the rectification of AMPA currents, but not so much for measuring the AMPA/NMDA ratios in figure 5B. As I see it, the AMPA/NMDA ratio is calculated to estimate the density of AMPA receptors in the synapse and thus indirectly estimate the strength of a synaptic connection. The ratio is indeed measured at +40 mV but this is only for technical reasons - the actual synaptic activity occurs at the resting membrane potential (RMP) of the cell and at this potential all AMPA receptors are fully activated (including the GluA2-lacking ones). By using spermine in the pipette, the authors miss the current contributed by GluA2-lacking AMPA receptors. Along this line, it is possible that in the non-devalued group the AMPA current would have been much bigger without spermine but not in the devalued group (I realize that that would contradict the lack of rectification, but still...).

2) A related question - to the naked eye it seems that the authors indeed saw rectification of the AMPA current in all groups. It even seems that the rectification is smaller in the devalued group. Can the authors test whether their data in the positive potentials is significantly different from the x=y line? And whether the non-devalued synapses are “more rectified” than the devalued cells?

3) All experiments were conducted in the light phase of the day, which is the less active and “appetitive” part of the day for mice. Are there any known effects of the circadian clock on striatal physiology in this context? Is it possible that contrasting effects found in previous studies may stem from the fact that the other studies were conducted during the dark phase?

4) Were all experiments conducted in the shell of the accumbens? If so, it would be good to be more specific and state that all these data are from the shell.

5) At times, it was a bit hard to follow in the Results all the interactions and triple interactions and their meaning.

6) The time line of the last 4 days in Figure 1A is not very clear in my opinion.

7) Figure 3B - I am not sure what is the benefit of showing the Alexa 568. I think it is obvious that if they see a GFP-expressing neurons they can patch it. What I think would be more helpful is to show a bright field image of the slice with at least two neurons, then the same view in GFP, and a merge showing that one of them is GFP+ and the other GFP- (just to clarify what is GFP+ and GFP- used throughout this figure).

8) Did the ANOVA on the number of spikes in GFP- neurons (Figure 3E) include also the 20, 32 and 44 steps? This could be a bit misleading. There may be a main devaluation effect in the GFP- cells that the authors are missing (for example, if the authors had used also 2, 4, 6, 8, 10, etc. steps up to 40 pA all of those would show no difference and would make all ANOVAs in this figure not significant, including 3C).

9) Line 113 - add University name.

10) Line 429 - I think the word “However” should be omitted.

11) Line 600 - the sentence needs to be re-writtten.

12) Figure 5 - rectification graphs in panel B are missing the x-axis title.

13) Figure 5 - what are the rectification values normalized to?
